# Re-evaluating colonoscopy screening intervals: extending the timeline to 15 years for low-risk individuals

**DOI:** 10.1097/JS9.0000000000001839

**Published:** 2024-06-20

**Authors:** Banwari L. Bairwa, Hamza Sajjad, Mahalaqua Nazli Khatib, Rakesh K. Sharma, Sarvesh Rustagi, Mahendra Pratap Singh, Ayush Anand

**Affiliations:** a MP Birla Hospital and Research Center, Chittorgarh, India; b MediSurg Research, Darbhanga, India; c Division of Evidence Synthesis, Global Consortium of Public Health and Research, Datta Meghe Institute of Higher Education, Wardha, India; d Graphic Era (Deemed to be University); e Graphic Era Hill University, Clement Town; f School of Applied and Life Sciences, Uttaranchal University, Dehradun, Uttarakhand, India; g Center for Global Health Research, Saveetha Medical College and Hospital, Saveetha Institute of Medical and Technical Sciences, Saveetha University, Chennai, India; h Medical Laboratories Techniques Department, AL-Mustaqbal University, Hillah, Babil, Iraq; i BP Koirala Institute of Health Sciences, Dharan, Nepal; j Frontier Medical College, Abbottabad, Pakistan

Colorectal cancer (CRC) is the third most common cancer globally and a significant cause of morbidity and mortality^1–3^. Early detection and removal of precancerous lesions through screening colonoscopies are critical strategies that have been proven to reduce the incidence and mortality of CRC^1,2^. The current guidelines generally recommend a 10-year interval between screening colonoscopies for individuals at average risk who have had a prior colonoscopy with negative findings^1,2,4^. However, this interval is not based on robust evidence from clinical trials, particularly for those with a completely negative initial screening result.

A recent large-scale cohort study provides compelling evidence that might change our current practice by extending the screening interval to 15 years for low-risk populations^5^. The study analyzed data from over 110 000 individuals who had an initial colonoscopy with negative findings for CRC (no adenomas, carcinoma in situ, or cancer) and compared them with ~1.98 million matched controls^5^. The median age of participants was 59 years, and they were followed for up to 29 years^5^. The major revelation was that the risks of CRC and CRC-specific mortality were significantly lower in the group with a negative first colonoscopy for up to 15 years compared to matched controls who did not undergo colonoscopy^5^. More specifically, after 15 years, the exposed group had a 10-year standardized incidence ratio of CRC at 0.72 and a mortality ratio of 0.55 compared to the control group^5^. This translates into a potential to miss only two CRC cases and one CRC-specific death per 1000 individuals screened if the interval between colonoscopies is extended from 10 to 15 years. This data suggests that the 10-year interval could be safely extended to 15 years in individuals with no family history of CRC and a negative initial screening colonoscopy.

These findings have profound implications for clinical practice (Table 1). First, extending the screening interval could significantly reduce the number of colonoscopies performed, thereby decreasing the risk of procedure-related complications, such as perforations and bleeding^6^. However, it should be noted that the risk of perforation and mortality during colonoscopy is low^7^. It also has the potential to improve the allocation of healthcare resources by increasing the availability of endoscopy services for higher-risk patients or those with symptoms indicative of CRC. Additionally, this change could reduce the psychological and physical burden on patients who currently undergo more frequent screening than might be necessary. This is particularly relevant given the invasive nature of colonoscopies and the preparation required, which many patients find uncomfortable^8^.

**Table 1:** Advantages and limitations of extending the colonoscopy screening interval.

Aspect	Advantages	Limitations and considerations
Clinical impact	Reduces risk of procedure-related complications like perforations and bleeding. Decreases psychological and physical burden on patients	Observational study design cannot establish causality. Does not apply to individuals with as family history of colorectal carcinoma
Healthcare resources	Improves availability of endoscopy services for higher-risk patients or symptomatic cases. Optimizes utilization of healthcare resources by reducing unnecessary screening	Since the study was conducted in Sweden, the findings may not be generalized globally
Screening efficiency	Misses only two colorectal carcinoma cases and one colorectal carcinoma-specific death per 1000 screened by extending interval	Advances in colonoscopy technology might influence detection rates
Patient experience	Reduces the frequency of invasive procedures and uncomfortable preparations for patients	Needs further research to validate findings in diverse populations
Policy implications	Can guide updates in clinical guidelines and recommendations	Longitudinal and cost-effectiveness studies needed for broader adoption

While the study is robust and its large sample size adds to the reliability of the findings, there are several limitations that must be acknowledged. First, the study is observational, which means it cannot establish causality. Second, the study was conducted in Sweden, and its findings may not be generalizable to populations with different demographics or healthcare systems, particularly in low-middle-income countries. Third, the study did not account for the potential impact of advancements in colonoscopy technology and technique, which could influence the detection rates of CRC and adenomas. Fourth, the study only included individuals with no family history of CRC, and therefore, its findings cannot be applied to those at higher risk. Fifth, the extension of the interval between colonoscopies may lead to missing CRC cases during screening. Hence, this should be validated in different settings to account for variabilities in reporting of CRC cases, and accordingly adjust the screening policies.

Integrating these findings into clinical practice should be done cautiously and in a manner that considers individual patient risk factors. For patients with a negative initial screening colonoscopy and no family history of CRC, clinicians could consider extending the interval for the next screening colonoscopy to 15 years. However, this should be a shared decision-making process, taking into account the patient’s preferences, overall health, and any other potential risk factors. It is also essential for guidelines to reflect these findings. Professional societies and regulatory bodies should consider updating their recommendations based on this new evidence to help standardize care and ensure patients receive the most appropriate, evidence-based care.

Further research is needed to validate these findings in different populations and healthcare settings. Longitudinal studies involving diverse populations will help determine if extending screening intervals can be safely recommended for various ethnic and racial groups who may have different baseline risks for CRC. Additionally, research into the cost-effectiveness of extending screening intervals would provide valuable information for policymakers and insurers. Studies that explore patient preferences and quality of life with different screening strategies are also needed to ensure that patient-centered care remains a priority. Finally, continued advancements in noninvasive screening methods, such as fecal immunochemical tests, could complement extended colonoscopy intervals, offering a potential strategy for maintaining high levels of screening while reducing the need for invasive procedures.

In conclusion, the interval between screening colonoscopies can be extended from 10 to 15 years in low-risk individuals. By adopting a more personalized approach to screening intervals, we can enhance patient care, optimize resource utilization, and maintain high standards of preventive health. However, these changes should be implemented thoughtfully, with ongoing research to further refine our understanding and ensure that all patients receive the most appropriate and effective screening.

## Ethical approval

Ethical approval is not applicable for this editorial article.

## Consent

Informed consent is not applicable for this editorial article.

## Source of funding

None.

## Author contribution

B.L.B. and H.S.: conceptualization, project administration, supervision, validation, writing – original draft, and writing – review and editing; M.N.K., R.K.S, M.P.S., and S.R.: writing – original draft and writing – review and editing; A.A.: conceptualization, project administration, supervision, validation, writing – original draft, and writing – review and editing.

## Conflicts of interest disclosure

The authors declares no conflicts of interest.

## Research registration unique identifying number (UIN)

None.

## Guarantor

Banwari Lal Bairwa.

## Data availability statement

Data sharing is not applicable to this article as no new data were created or analyzed in this study.

## Provenance and peer review

Not commissioned, externally peer-reviewed.

## Assistance with the study

None.

## Presentation

None.
